# AGEs Inducing EPCs Apoptosis via ROS and p38 MAPK/JNK Pathways in Diabetic Vascular Complications

**DOI:** 10.33549/physiolres.935554

**Published:** 2025-08-01

**Authors:** Xi YANG, Zhi ZHU, Zihui LI, Xinlian SI, Lingyu KONG, Jiaming LIU, Lu YANG, Huqiang HE

**Affiliations:** 1 Department of Vascular Surgery, The Affiliated Hospital of Southwest Medical University, Luzhou, China; 2 The Center of Vascular and Interventional Surgery, Department of General Surgery, The Third People’s Hospital of Chengdu & The Affiliated Hospital of Southwest Jiaotong University, Chengdu, China

**Keywords:** Advanced glycation end products, Endothelial progenitor cells, Oxidative stress, Cell apoptosis, p38 MAPK/JNK

## Abstract

Endothelial progenitor cells (EPCs) promote blood-vessel repair, but their apoptosis worsens diabetes-related vascular damage. Although advanced glycation end products (AGEs) abound in diabetes, it remains unclear whether they trigger EPC apoptosis through oxidative stress and driven MAPK activation. EPCs were extracted from Sprague-Dawley rats’ bone marrow. Cells were treated with varying AGEs concentrations (50, 100, 200 μg/mL) and durations (6, 12, 24, 48 hours). Apoptosis was measured *via* Annexin V/PI staining flow cytometry, and protein expression of Bax (pro-apoptotic) and Bcl-2 (anti-apoptotic) was analyzed by Western blot. Reactive oxygen species (ROS) levels were detected by flow cytometry using DCFH-DA molecular probes. The effects of antioxidants (NAC) and specific inhibitors (SP600125 for JNK and SB203580 for p38MAPK) on apoptosis and protein expression were also examined. EPC apoptosis increased with AGEs concentration and exposure time, peaking at 24 hours. Bax expression rose, while Bcl-2 decreased with higher AGEs levels and prolonged exposure. ROS generation increased up to 12 hours before declining. Antioxidant NAC reduced ROS and Bax expression. Inhibitors SP600125 and SB203580 decreased JNK and p38MAPK activation, lowered Bax, and increased Bcl-2 expression.

AGEs induce EPC apoptosis through oxidative stress and the MAPK pathway; antioxidants or JNK/p38 inhibitors therefore warrant exploration to preserve EPC function in diabetes.

## Introduction

Diabetes mellitus (DM) is a chronic metabolic disorder characterized by persistent hyperglycemia, and it remains a leading cause of morbidity and mortality globally. Among its complications, diabetic macro- and microvascular pathologies are significant contributors to disability and death, primarily driven by endothelial dysfunction and vascular damage [1,2]. Circulating endothelial progenitor cells (EPCs), derived from bone marrow, are essential for vascular repair and neovasculogenesis, playing a pivotal role in maintaining vascular homeostasis [3–5]. In diabetes, EPC dysfunction contributes significantly to the pathogenesis of diabetic vasculopathy. A recent meta-analysis demonstrated that reduced EPC levels are strongly associated with increased risks of cardiovascular events, cardiovascular mortality, and all-cause mortality in diabetic patients [6].

Advanced glycation end products (AGEs), a group of senescent macroprotein derivatives formed at an accelerated rate in hyperglycemic environments, have been identified as critical mediators in the progression of diabetic complications [7,8]. AGEs contribute to vascular damage by interacting with their primary receptor, RAGE (receptor for advanced glycation end products), which activates a cascade of downstream signaling pathways. In particular, AGEs/RAGE signaling has been shown to exacerbate endothelial injury by inducing EPC apoptosis, further impairing endothelial repair mechanisms [9]. Previous studies in osteoblasts and fibroblasts have revealed that AGEs induce the production of reactive oxygen species (ROS) and activate mitogen-activated protein kinase (MAPK) pathways, including JNK and p38 MAPK, promoting cellular apoptosis [10,11]. Seeger *et al*. showed that p38 MAPK activation alone can reduce EPC survival [12]. However, the specific mechanisms by which AGEs and ROS influence MAPK signaling to mediate EPC apoptosis in diabetic vasculopathy remain insufficiently explored.

Oxidative stress, primarily driven by excessive ROS production, is a key contributor to EPC dysfunction in diabetes [13,14]. AGEs-triggered ROS not only damage cellular components but also act as signaling molecules to activate pro-apoptotic pathways such as JNK and p38 MAPK [15]. These pathways are crucial mediators of cellular apoptosis, particularly in conditions of sustained oxidative stress. While evidence suggests that modulation of ROS levels and inhibition of MAPK signaling may protect EPCs from apoptosis, further research is needed to elucidate the temporal dynamics of ROS production and its interplay with MAPK signaling in EPC apoptosis[16].

This study aimed to investigate the mechanisms by which AGEs induce EPC apoptosis, focusing on ROS production and the activation of the p38 MAPK/JNK signaling axis. We examined the concentration- and time-dependent effects of AGEs on EPC apoptosis and analyzed the role of ROS and MAPK pathways using specific inhibitors. Our findings provide insights into potential therapeutic targets for mitigating diabetic vascular complications by preserving EPC function and reducing apoptosis through the modulation of oxidative stress and MAPK pathway activity.

## Methods

All animal experiments in this study were conducted in strict accordance with institutional and national guidelines for the care and use of laboratory animals. The protocol was approved by the Animal Ethics Committee of The Affiliated Hospital of Southwest Medical University (No. 24101115).

### Preparation and isolation of rat bone marrow mononuclear cells

Sprague-Dawley rats (4 months, 170 g ± 10 g) were selected and humanely euthanized before being disinfected through immersion in 75 % ethanol for 20 minutes and placed on a sterile, UV-irradiated bench. The femur and tibia were surgically removed using sterile instruments and the bone marrow was harvested by flushing with 1 % heparinized PBS until the bones turned white, collecting the effluent in 10 mL centrifuge tubes. This bone marrow wash was then centrifuged at 1500 rpm at room temperature for 5–10 minutes to sediment the cells, which were subsequently re-suspended in cold PBS and carefully layered over lymphocyte separation medium (Ruibo Biotechnology, Guangzhou, China) for density gradient centrifugation at 2000 rpm for 25 minutes, resulting in a layered separation of cellular components. The mononuclear cell layer was isolated, washed to remove impurities, and resuspended in EGM-2 complete culture medium (Lonza, Houston, United States) to a concentration of 1×10^6^ cells/mL. These cells were then cultured in six-well plates with fresh medium and incubated under standard conditions, establishing a foundation for subsequent experimental manipulations and analyses.

### Culture of rat bone marrow-derived EPCs

EPCs were isolated from rat bone marrow and cultured in six-well plates with EGM-2 complete culture medium. Starting from day three, the culture medium was meticulously replaced every three days after washing non-adherent cells with sterile, cold PBS. After each medium change, cell growth and differentiation were examined under an inverted phase-contrast microscope. For the identification of EPCs, after seven days of culture, cells were first washed with PBS and then incubated with 10 μg/mL DiI-acetylated low-density lipoprotein (DiI-acLDL, Thermo Fisher Scientific, Waltham, United States) for one hour at 37 °C. Following the incubation, cells were washed multiple times with PBS and fixed in 4 % paraformaldehyde (GenScript, Nanjing, China). Subsequently, cells were incubated with 10 μg/mL FITC-labeled Ulex europaeus agglutinin I (FITC-UEA-1, Sigma, Michigan, United States) for an additional 90 minutes at 37 °C. After final washes, the cells were examined under a fluorescence microscope, where EPCs demonstrated dual red and yellow fluorescence by successfully engulfing DiI-acLDL and binding FITC-UEA-1, indicative of their differentiation and identification as EPCs.

### Intervention of rat EPCs with AGE-BSA

EPCs at passages 3–10 were cultured in six-well plates and monitored regularly until cell confluency reached 90 %, marking the initiation of the experimental model intervention. The existing medium was discarded, and the cells were gently washed with sterile PBS for approximately one minute. This was followed by the replacement of the medium with serum-free culture medium to induce a state of starvation in the cells for 24 hours. Subsequently, culture media with predetermined concentrations of AGE-BSA (Abcam, Cambridge, United Kingdom) were added to the cells, which were then cultured for additional periods of 6, 12, 24, and 48 hours, respectively. After these incubation periods, the cells were harvested for subsequent molecular biology assays.

### Flow cytometry analysis of EPC apoptosis using Annexin-FITC and PI double staining

The apoptosis rates of EPCs were systematically determined after treatments with AGEs at concentrations of 0 μg/mL, 50 μg/mL, 100 μg/mL, and 200 μg/mL, and following exposure to 200 μg/mL AGEs for periods of 6, 12, 24, and 48 hours [17]. This analysis extended to EPCs preconditioned with 20 μM SP600125 (Selleck, Houston, United States) and 20 μM SB203580 (Selleck, Houston, United States) before being subjected to 200 μg/mL AGEs for 24 hours [18,19]. EPCs harvested after AGE treatment underwent cell density adjustment, repeated washing, and resuspension for staining with Annexin-FITC/PI (SinoBiological, Beijing, China), followed by flow cytometry analysis within 30 minutes. The resulting apoptosis profiling via flow cytometry delineated early apoptotic (Q4), late apoptotic or necrotic (Q2), and healthy cells (Q1) across four quadrants. The rate of apoptosis was inferred from the cell distribution within the early and late apoptotic quadrants, offering a precise quantification of apoptosis across a range of AGE exposure scenarios, thereby enhancing our understanding of EPCs’ susceptibility to AGE-induced apoptotic triggers.

### Detection of ROS levels in EPCs using DCFH-DA

The intracellular ROS levels within EPCs were quantitatively assessed using 2.5 μM 2′,7′-dichlorofluorescein diacetate (DCFH-DA, Absin, Shanghai, China), a fluorescent probe sensitive to oxidative stress conditions. Initially non-fluorescent, DCFH-DA becomes fluorescent upon reaction with ROS, providing a direct measure of oxidative stress within the cells. EPCs were subjected to AGEs at a concentration of 200 μg/mL for durations of 3, 6, 12, and 24 hours, alongside control groups treated with 20 μM N-acetylcysteine (NAC) for antioxidative comparison and a positive control using H_2_O_2_. Following treatment, EPCs were collected and washed with PBS. Cells were washed three times with PBS to remove excess probe and then resuspended in 500 μL of PBS for immediate analysis. The fluorescence intensity of DCF, indicative of ROS levels, was measured using flow cytometry, allowing for a precise evaluation of the oxidative stress response in EPCs under different experimental conditions.

### Quantitative Western Blot analysis of signal pathway activation and apoptotic response in AGEs-Treated Endothelial Progenitor Cells

Western blotting was employed to measure the expression levels of Bax, Bcl-2, P-p38MAPK, T-p38MAPK, P-JNK, and T-JNK in EPCs following AGEs treatment at 50, 100, and 200 μg/mL concentrations over 6, 12, 24, and 48 hours, as well as after the application of 20 μM SP600125 and 20 μM SB203580 inhibitors post-24-hour AGEs exposure. EPCs, upon reaching 90 % confluency in 6-well plates, were washed with PBS chilled to 4 °C and lysed in RIPA buffer (Servicebio, Wuhan, China) enhanced with protease inhibitors. The cell lysates underwent sonication, followed by centrifugation at 12,000 g for 5 minutes at 4 °C to separate the supernatant containing the total protein. Protein separation was achieved through SDS-PAGE (Servicebio, Wuhan, China) using 8 %, 10 %, or 12 % gels based on target protein size, and subsequently, proteins were transferred to PVDF membranes. These membranes were blocked with 5 % skim milk in TBST (Servicebio, Wuhan, China) for one hour at room temperature, incubated overnight at 4 °C with primary antibodies diluted 1:500 against target proteins (all from Cell Signaling Technology, Boston, United States), washed, and incubated with HRP-conjugated secondary antibodies (Proteintech, Wuhan, China) diluted 1:2000 for one hour at room temperature. After thorough washing, the protein bands were visualized using enhanced chemiluminescence. Band intensities were quantified using ImageJ software, normalizing against GAPDH (Servicebio, Wuhan, China) to calculate relative protein expression, facilitating comparative analysis among different treatments and conditions.

### Statistics

The data obtained from these measurements and analyses were processed and analyzed using SPSS version 26.0. The relative fluorescence intensity values of DCFH-DA, the relative grayscale values of Western blot protein bands, and the apoptosis rates determined by flow cytometry are all presented as mean ± standard deviation (SD). A t-test was employed to compare differences between two groups, while one-way analysis of variance (ANOVA) was utilized for the analysis of differences among multiple groups. A p-value of less than 0.05 was considered statistically significant.

## Results

### Morphological changes and identification of EPCs

**Fig. 1:**
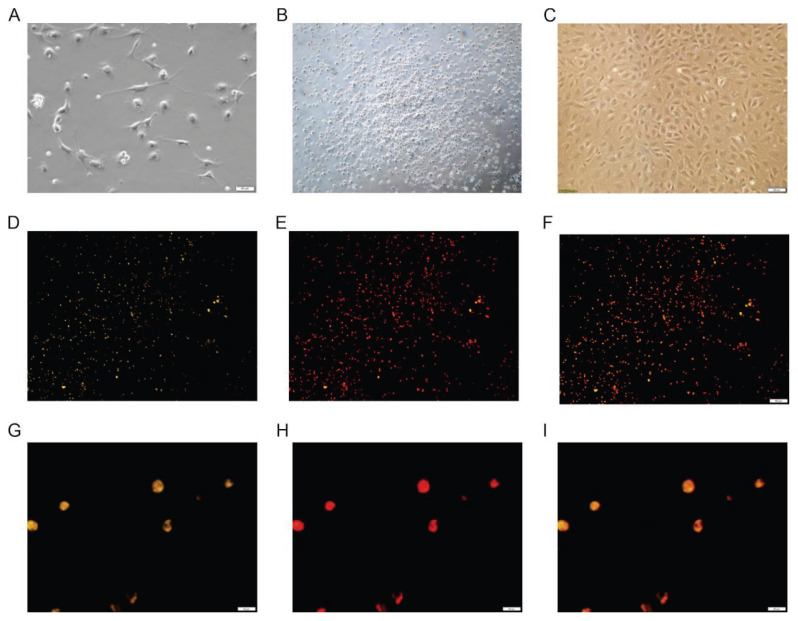
Morphological changes and identification of EPCs during cultivation. A–C: Phase-contrast images showing morphological evolution on day 3 (**A**), day 7 (**B**) and day 14 (**C**). Scale bar = 100 μm. D–F: Low-magnification (200×) fluorescence micrographs of EPCs on day 7 after double staining with Dil-acLDL (red) and FITC-UEA-1 (green). Scale bar = 100 μm. G–I: High-magnification (400 ×) confocal images of the same day-7 culture, illustrating co-localisation of Dil-acLDL (red) and FITC-UEA-1 (green). Scale bar = 50 μm.

EPCs displayed distinct morphological changes over the course of cultivation. By day 3, adherent cells were observed under the microscope, predominantly round in shape, with a smaller population exhibiting elliptical, fusiform, or spindle morphologies (Fig. 1A). By day 7, the cells underwent rapid growth, and elliptical and spindle shapes became more prominent, forming initial colony-like clusters (Fig. 1B). By day 14, the cells developed typical cobblestone and vortex-like growth patterns, characteristic of EPCs (Fig. 1C). Identification of EPCs was confirmed using dual fluorescent staining with Dil-acLDL and FITC-UEA-1. Low-magnification (200×) fluorescence images demonstrated that the great majority of cells were double positive (Fig. 1D–F), confirming that marker expression was not confined to isolated cells. High-magnification confocal images (400×) further verified co-localisation of Dil-acLDL and FITC-UEA-1 within individual cells (Fig. 1G–I).

### EPC apoptosis induced by AGEs-BSA

**Fig. 2:**
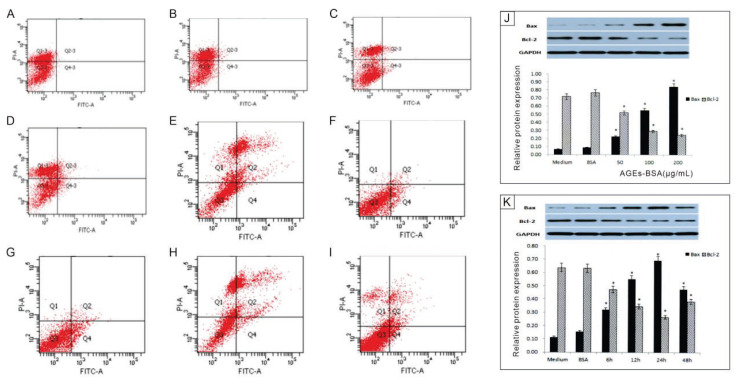
Effects of AGEs-BSA concentration and treatment duration on EPC apoptosis and apoptosis-related protein expression. A–E: Apoptosis rates of EPCs treated with medium (**A**), Con+BSA (**B**), AGEs-BSA 50 μg/mL (**C**), AGEs-BSA 100 μg/mL (**D**), and AGEs-BSA 200 μg/mL (**E**) for 24 hours. F–I: Time-dependent apoptosis rates of EPCs treated with 200 μg/mL AGEs-BSA for 6 hours (**F**), 12 hours (**G**), 24 hours (**H**), and 48 hours (**I**). J–K: Western blot analysis of Bax and Bcl-2 protein expression in EPCs (n = 3).

AGEs-BSA treatment significantly increased apoptosis in EPCs in a concentration- and time-dependent manner. Apoptosis rates in the control groups (Medium and Con+BSA) were low, at 2.92 % and 3.15 %, respectively (Fig. 2A, B). Following treatment with AGEs-BSA at 50, 100, and 200 μg/mL for 24 hours, apoptosis rates increased to 5.94 %, 9.83 %, and 24.81 %, respectively (Fig. 2C–E). A time-course analysis using 200 μg/mL AGEs-BSA revealed that apoptosis rates increased progressively over time, reaching 15.83 %, 22.75 %, and 27.86 % at 6, 12, and 24 hours, and slightly declining to 23.24 % at 48 hours (Fig. 2F–I). These findings indicate that AGEs-BSA induces maximal apoptosis at 24 hours, with a subsequent plateau phase.

Western blot analysis showed a dose- and time-dependent increase in Bax protein expression, a pro-apoptotic marker, and a corresponding decrease in Bcl-2, an anti-apoptotic protein (Fig. 2J, K). These results suggest that AGEs-BSA-mediated apoptosis involves dysregulation of the Bax/Bcl-2 ratio, favoring apoptotic signaling in EPCs.

### Oxidative stress induced by AGEs-BSA and its modulation by NAC

**Fig. 3:**
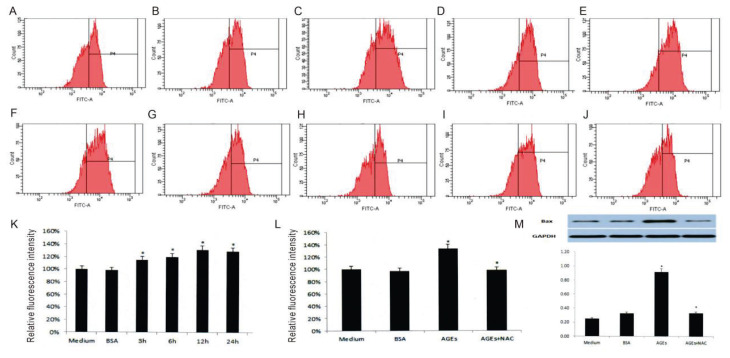
AGEs-BSA induces oxidative stress and apoptosis in EPCs, attenuated by NAC pretreatment. A–F: EPCs were treated with medium (**A**), Con+BSA (**B**), or AGEs-BSA (200 μg/mL) for 3 hours (**C**), 6 hours (**D**), 12 hours (**E**), and 24 hours (**F**). G–J: EPCs were treated with medium (**G**), Con+BSA (**H**), AGEs-BSA for 24 hours (**I**), and AGEs-BSA with NAC pretreatment (20 μM) (**J**). K–L: Quantification of DCFH-DA fluorescence. **M**) Western blot analysis of Bax expression (n = 3). H_2_O_2_ was used for assay verification but omitted from the graph to maintain scale.

AGEs-BSA significantly elevated ROS production in EPCs, as detected by DCFH-DA fluorescence intensity. ROS levels increased with time, reaching 114.41 %, 119.49 %, 131.36 %, and 128.32 % of the control group at 3, 6, 12, and 24 hours, respectively, peaking at 12 hours (Fig. 3A–F). These results demonstrate that AGEs-BSA triggers oxidative stress early during exposure, contributing to apoptosis.

Pretreatment with the antioxidant NAC (20 μM) markedly reduced ROS levels, as indicated by fluorescence returning to near-control levels (98.21 % *vs*. 133.51 %, P<0.01, Fig. 3G–J). Western blot analysis further revealed that NAC pretreatment significantly downregulated Bax expression (0.86±0.01 *vs*. 0.24±0.01, P<0.01), confirming its role in mitigating AGEs-BSA-induced oxidative stress and apoptosis (Fig. 3K–M). These findings indicate that ROS generation plays a pivotal role in AGEs-BSA-induced EPC apoptosis, and antioxidant intervention can reverse this process.

### Activation of p38 MAPK and JNK by AGEs-BSA

**Fig. 4:**
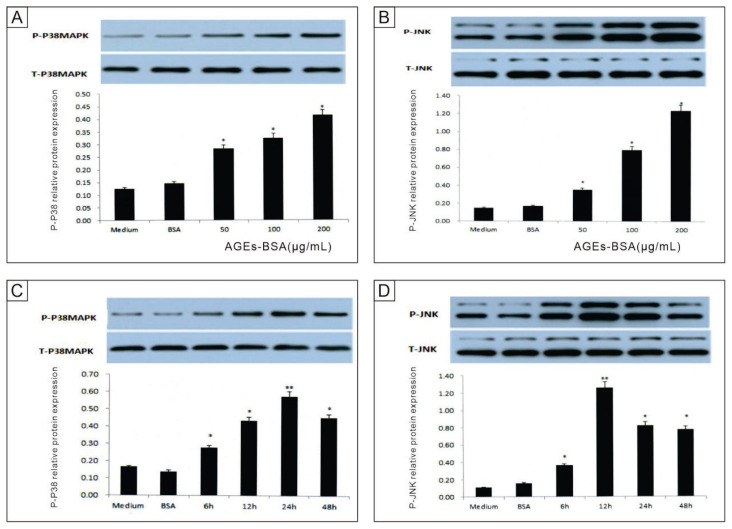
Effects of AGEs-BSA concentration and treatment duration on P-p38MAPK and P-JNK protein expression in EPCs. A–B: P-p38MAPK (**A**) and P-JNK (**B**) protein expression levels in EPCs after treatment with different concentrations of AGEs-BSA (0 μg/mL, 50 μg/mL, 100 μg/mL, 200 μg/mL) for 24 hours. C–D: Time-dependent expression of P-p38MAPK (**C**) and P-JNK (**D**) in EPCs treated with 200 μg/mL AGEs-BSA for 6, 12, 24, and 48 hours (n = 3).

AGEs-BSA treatment activated the MAPK signaling pathways, specifically JNK and p38 MAPK, in a concentration- and time-dependent manner. With increasing AGEs-BSA concentrations (50, 100, 200 μg/mL), P-p38MAPK expression increased significantly, from 0.28±0.01, 0.33±0.01 to 0.41±0.02 (P<0.01, Fig. 4A). Similarly, P-JNK levels rose from 1.08±0.02, 1.36±0.01 to 1.75±0.01 (P<0.01, Fig. 4B). A time-course analysis revealed that P-JNK expression peaked at 12 hours and subsequently declined, while P-p38MAPK expression progressively increased, peaking at 24 hours (Fig. 4C, D). Notably, total p38MAPK and JNK levels remained unchanged, indicating that the observed effects were driven by phosphorylation events. These results suggest that AGEs-BSA induces oxidative stress, leading to the activation of JNK and p38 MAPK pathways, which mediate EPC apoptosis.

### Effects of JNK and p38MAPK inhibitors on AGEs-BSA-induced apoptosis

**Fig. 5:**
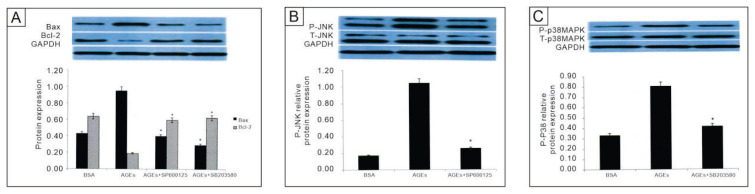
Effects of JNK and p38MAPK inhibitors on AGEs-BSA-induced apoptosis and related protein expression in EPCs. **A**) Western-blot analysis of Bax and Bcl-2. Treatment groups: control + BSA, AGEs (200 μg/mL, 24 h); AGEs + JNK inhibitor SP600125 (20 μM, 1 h pretreatment) and AGEs + p38 MAPK inhibitor SB203580 (20 μM, 1 h pretreatment). **B**) Phospho- and total JNK (P-JNK, T-JNK) in control + BSA, AGEs, and AGEs + SP600125 groups. **C**) Phospho- and total p38 MAPK (P-p38, T-p38) in control + BSA, AGEs, and AGEs + SB203580 groups. *P < 0.05 *vs*. control + BSA. (n = 3).

To further elucidate the role of JNK and p38 MAPK in AGEs-BSA-induced apoptosis, EPCs were pretreated with the JNK inhibitor SP600125 and the p38 MAPK inhibitor SB203580. Pretreatment with SP600125 significantly reduced P-JNK levels (1.05±0.01 to 0.25±0.01, P<0.01) and Bax expression (0.95±0.01 to 0.40±0.01, P<0.01), while increasing Bcl-2 levels (0.19±0.01 to 0.59±0.01, P<0.01, Fig. 5 A, B). Similarly, SB203580 pretreatment significantly down-regulated P-p38MAPK (0.80±0.01 to 0.41±0.02, P<0.01) and Bax expression (0.95±0.01 to 0.29±0.01, P<0.01), while upregulating Bcl-2 (0.19±0.01 to 0.62±0.01, P<0.01, Fig. 5 A, C). These findings confirm that JNK and p38 MAPK are critical mediators of AGEs-BSA-induced apoptosis in EPCs. Both pathways are activated downstream of ROS generation and contribute to pro-apoptotic signaling by modulating Bax/Bcl-2 expression. Inhibiting these pathways effectively reduces apoptosis, highlighting their potential as therapeutic targets for mitigating diabetic vascular complications.

## Discussion

This study provides critical insights into the mechanisms by which AGEs induce EPC apoptosis and highlights the roles of oxidative stress and the p38 MAPK/JNK pathways in the progression of diabetic vascular complications. Our findings contribute to the growing body of evidence underscoring the significance of EPC dysfunction in diabetes-related vascular injury and identify potential therapeutic targets to mitigate these complications.

EPCs, first identified by Asahara *et al*. in 1997 as CD34+ and KDR+ cells derived from bone marrow, are crucial for vascular homeostasis and repair through their role in neovascularization and damaged vessel repair [20]. They are primarily sourced from bone marrow, peripheral blood, and umbilical cord blood, with bone marrow being the most abundant source. EPC dysfunction has been implicated in a wide range of pathologies, particularly diabetes and atherosclerosis, where reduced EPC number and functionality exacerbate vascular injury [21,22]. Our findings reinforce this concept by demonstrating that AGEs induce EPC apoptosis in a concentration- and time-dependent manner. This evidence adds mechanistic clarity to previous studies that reported decreased EPC survival and functionality in diabetes.

AGEs, formed at an accelerated rate in hyperglycemic environments, interact with their receptor RAGE to mediate various downstream pathological effects. In this study, we found that AGEs significantly elevated EPC apoptosis by altering the balance of pro-apoptotic (Bax) and anti-apoptotic (Bcl-2) proteins. This imbalance highlights a critical mechanism by which AGEs disrupt EPC survival. Consistent with prior studies, the AGEs-RAGE axis has been shown to inhibit EPC proliferation, migration, tube formation, and adhesion, ultimately impairing vascular repair and exacerbating diabetic vascular complications [23–25]. These findings underscore the importance of managing AGEs accumulation in diabetic patients to preserve EPC function and maintain vascular homeostasis.

One of the key findings of this study is the central role of oxidative stress, mediated by ROS, in AGE-induced EPC apoptosis. ROS levels increased significantly in response to AGEs, peaking at 12 hours, and were associated with increased Bax expression and apoptosis. ROS, as secondary messengers, are well-established mediators of oxidative stress-induced cellular dysfunction [26,27]. Treatment with the antioxidant NAC effectively reduced ROS levels, decreased Bax expression, and restored Bcl-2 expression, confirming that oxidative stress is a pivotal driver of AGE-induced apoptosis. These results are consistent with prior studies that identified oxidative stress as a major contributor to diabetic vascular complications, suggesting that targeting ROS production may serve as a viable therapeutic strategy [28]. ROS can activate NF-κB, which in turn up-regulates Bax and augments JNK/p38 signalling, while prolonged oxidative stress may trigger compensatory autophagy that dampens mitochondrial damage yet, if overwhelmed, feeds forward to p38-mediated cell death [29,30].

In addition to ROS, we demonstrated that AGEs activate the p38 MAPK and JNK pathways, which are critical mediators of pro-apoptotic signaling. The activation of these pathways was time-dependent, with P-p38MAPK peaking at 24 hours and P-JNK peaking at 12 hours. Inhibitors of these pathways, SP600125 (JNK) and SB203580 (p38 MAPK), significantly reduced Bax expression and increased Bcl-2 expression, confirming the involvement of these pathways in AGE-induced EPC apoptosis. These findings align with prior studies showing that the MAPK pathway is a central mediator of AGE-induced cellular apoptosis [16,31]. The identification of these pathways in EPCs extends our understanding of their role in diabetic vascular complications and suggests that pharmacological inhibition of p38 MAPK and JNK could protect EPCs from apoptosis.

While earlier studies have explored AGE-induced apoptosis in other cell types, such as fibroblasts [11,32] and osteoblasts [17,33], this study extends those findings to EPCs, a critical cell population in vascular repair. By elucidating the interplay between ROS, p38 MAPK, and JNK signaling in EPCs, we provide a clearer understanding of how AGEs impair vascular repair mechanisms in diabetes. Notably, the observed dynamics of ROS and MAPK activation in EPCs suggest a potential therapeutic window for intervention, where timely administration of antioxidants or MAPK inhibitors could prevent irreversible cellular damage.

Our findings have important clinical implications. The dual approach of targeting ROS production and MAPK signaling pathways offers a promising strategy to preserve EPC function and mitigate vascular injury in diabetes. Antioxidants such as NAC, which reduce oxidative stress, combined with pharmacological inhibitors of MAPK pathways, could synergistically protect EPCs from apoptosis. Petrelli *et al*. reported pharmacological p38 MAPK inhibitors and other drugs can rescue EPC function under diabetic conditions [34]. The antioxidant lycopene was also found to inhibit high glucose/AGE-induced EPC apoptosis by sup-pressing ROS generation and p38 MAPK activation [35]. However, further research is needed to validate the efficacy of these interventions in preclinical and clinical settings. For example, animal models of diabetic vascular complications could be used to assess the long-term effects of combined antioxidant and MAPK inhibitor therapies on vascular outcomes.

This study also exsits some limitations. First, EPC identity was evaluated solely by Dil-acLDL/FITC-UEA-1 staining without the flow-cytometric quantification of CD34, CD133, and VEGFR-2 that is standard for rigorous phenotyping. Second, all experiments were performed in rat EPC monocultures devoid of haemodynamic shear, immune mediators, and systemic antioxidant buffering; such inter-species and microenvironmental differences constrain direct extrapolation to human diabetic vasculopathy. Third, the glycation insult was modelled with a single purified AGE-BSA species, whereas diabetic plasma contains a chemically diverse array of AGEs and additional metabolic stressors. Accordingly, the contribution of ROS-driven JNK/p38 activation to EPC attrition has yet to be quantified.

In conclusion, this study provides compelling evidence that AGEs induce EPC apoptosis through ROS production and activation of the p38 MAPK and JNK signaling pathways, contributing to endothelial injury in diabetic vasculopathy. These findings enhance our understanding of the molecular mechanisms underlying diabetic vascular complications and suggest potential therapeutic strategies to protect EPCs and improve vascular outcomes. Future studies should focus on developing combination therapies targeting oxidative stress and MAPK pathways and evaluating their efficacy in preventing or reversing diabetic vascular complications.

## Data Availability

The datasets and materials used and/or analyzed during the current study are available from the corresponding author upon reasonable request.
